# Hostility and self-destructive behavior in depression

**DOI:** 10.1192/j.eurpsy.2025.1305

**Published:** 2025-08-26

**Authors:** T. I. Medvedeva, O. M. Boyko, S. N. Enikolopov, O. Y. Vorontsova

**Affiliations:** 1 Clinical psychology, Mental Health Research Center, Moscow, Russian Federation

## Abstract

**Introduction:**

Self-destructive behavior in depression is most often a pathological way of emotional self-regulation, but it may increase suicidal risk. In this regard, the identification of risk factors for self-destructive behavior is of practical importance.

**Objectives:**

The aim was to study hostility as risk factor self-destructive behavior in depression.

**Methods:**

The study included 155 women from 16 to 25 years old (mean age 19,77) hospitalized with depression in a clinic of FSBSI MHRC. The Buss-Perry Aggression Questionnaire (BPAQ), the Symptom Checklist-90-reserved (SCL-90-R), the Physical Appearance Comparison Scale—Revised (PACS–R), **t**he Body Investment Scale (BIS), anamnestic data about self-destructive behavior, a question to assess the level of autoaggression («Sometimes I deliberately injure myself » was measured on a Likert scale from 0 to 5) were used. ANOVA and Spearman’s rank correlation coefficient were used. To analyze the features associated with the level of hostility, the sample was divided into subgroups “with low hostility” (78 people, mean age 19.91) and “with high hostility” (77 people, mean age 19.6), according to the median value of the “Hostility” parameter of BPAQ - 23.

**Results:**

Correlation analysis showed a statistically significant relationship between the level of “Hostility” and the frequency of “deliberately injure” (r=0.19, p<0.05). Comparison of subgroups according to the parameters of SCL-90-R showed a higher level of psychopathologic symptomatology in the group with high hostility: SOM (1.11±0.80 in the “low hostility” group vs. 1.50±0.85 in the “high hostility” group at p=0.001), OC (1.60±0.87 vs. 2, 16±0.77 at p=0.000), INT (1.32±0.84 vs. 2.16±0.90 at p=0.000), DEP (1.73±0.98 vs. 2.27±0.87 at p=0.000), ANX (1.27±0.92 vs. 1.90±1.01 at p=0.000), HOS (0, 89±0.87 vs. 1.41±0.89 at p=0.000), PHOB (0.81±0.86 vs. 1.42±0.89 at p=0.000), PAR (0.74±0.65 vs. 1.43±0.84 at p=0.000), PSY (0.87±0.65 vs. 1, 26±0.78 at p=0.000), GSI (1.22±0.66 compared to 1.77±0.71 at p=0.000), PDSI (2.12±0.60 compared to 2.39±0.56 at p=.002), PSI (48.66±17.14 compared to 64.20±16.16 at p=0.000). BIS scores on the “body Investment subscale” (3.18±1.18 compared to 2.55±1.12 at p=0.001) on the total scale (3.38±0.58 compared to 3.15±0.54 at p=0.008) The PACS-R scale data demonstrate differences in the physical comparison parameter (2.02±1.37 versus 2.66±1.23 at p=0.002).

**Conclusions:**

A higher level of hostility in depression in women with self-destructive behavior is associated with both a worse mental status and a stronger rejection of one’s body, manifested in a decreased concern for it and an increased need to compare oneself with others. This allows us to consider hostility as one of the markers of high risk of self-harming behavior in depression.

**Disclosure of Interest:**

None Declared

